# Journal data policies: Exploring how the understanding of editors and authors corresponds to the policies themselves

**DOI:** 10.1371/journal.pone.0230281

**Published:** 2020-03-25

**Authors:** Thu-Mai Christian, Amanda Gooch, Todd Vision, Elizabeth Hull

**Affiliations:** 1 Odum Institute for Research in Social Science, University of North Carolina at Chapel Hill, Chapel Hill, North Carolina, United States of America; 2 Department of Biology, University of North Carolina at Chapel Hill, Chapel Hill, North Carolina, United States of America; 3 Dryad, Durham, North Carolina, United States of America; Indiana University Bloomington, UNITED STATES

## Abstract

Despite the increase in the number of journals issuing data policies requiring authors to make data underlying reporting findings publicly available, authors do not always do so, and when they do, the data do not always meet standards of quality that allow others to verify or extend published results. This phenomenon suggests the need to consider the effectiveness of journal data policies to present and articulate transparency requirements, and how well they facilitate (or hinder) authors’ ability to produce and provide access to data, code, and associated materials that meet quality standards for computational reproducibility. This article describes the results of a research study that examined the ability of journal-based data policies to: 1) effectively communicate transparency requirements to authors, and 2) enable authors to successfully meet policy requirements. To do this, we conducted a mixed-methods study that examined individual data policies alongside editors’ and authors’ interpretation of policy requirements to answer the following research questions. Survey responses from authors and editors along with results from a content analysis of data policies found discrepancies among editors’ assertion of data policy requirements, authors’ understanding of policy requirements, and the requirements stated in the policy language as written. We offer explanations for these discrepancies and offer recommendations for improving authors’ understanding of policies and increasing the likelihood of policy compliance.

## Introduction

Journals play a pivotal role in academic incentive structures, and thus have the power to act as catalysts for change in research practices that proponents of open science advocate [1]. Indeed, Vines et al. [2] found a correlation between the strength of a journal-issued data policy and the availability of data associated with articles published in those journals. Moreover, results from a study conducted by Kim and Adler [3] showed that journal-issued data policies have a statistically significant positive effect on data sharing behaviors. Journals have taken note of this.

As of October 2018, almost 5,000 journals have added their names to the growing list of signatories of the Transparency and Openness Promotion (TOP) Guidelines published by the Center for Open Science. As signatories, these journals have declared “their support of the principles of openness, transparency, and reproducibility, expressing interest in the guidelines and commit to conducting a review within a year of the standards and levels of adoption” [4]. The TOP Guidelines are unique in that they offer journals a framework with which to operationalize these principles in standardized policies for data, analytic methods, and materials transparency that can be implemented at varying levels of stringency—from requiring authors to submit a declaration of the accessibility of data, code, and research materials used to produce reported results (TOP Level I), to requiring deposit of these research artifacts in a trusted repository (TOP Level II), to requiring an independent verification of computational reproducibility using the artifacts to reproduce reported results (TOP Level III). Major publishers, including Taylor & Francis [5], Elsevier [6], and Springer Nature [7] have all expressed support for the TOP Guidelines, which is reflected in these publishers’ tiered data policy structures they subsequently established for adoption by their journals.

Recent studies that investigated the impact of data policies on data access and quality found that, even with a data policy imposed upon them, authors are not always making their underlying data available, and when they do, the data frequently do not meet standards of quality that allow for verification of results [8–9]. One problem may be that those policies lack the necessary strength to compel compliance because requirements are not strictly stated and/or failure to comply do not result in any sanctions.

Based on recent studies that have examined the content of the policies of highly rated journals, however, only a small handful achieve the stringency of at least a TOP Level II policy [10–11] that requires authors to submit their data, code, and other associated research materials to a trusted repository. In Piwowar and Chapman’s study on the impact of journal policies on data sharing, journals were classified into those having no policy, a weak policy (i.e., suggestion or ill-defined requirement) or a strong policy (i.e., well-described requirement). They found that the strong policies yielded higher median rates of data sharing [12]. In their consideration of policies categorized by strength, Stodden, Guo, & Ma noted that without “clearly and prominently [stating] (in the instructions for authors and on their Web sites) their policies…and the consequences for authors who do not adhere,” policies less effectively encourage compliance [13].

TOP Guidelines consider transparency standards to be implemented only when requirements are in place, with encouragement considered to be non-implementation. In comparing this with classifications of journals based on strength of requirements used in previous studies, policies that adopt any TOP level beyond Level 0 (“non-implementation”) are considered to be at the highest levels of stringency and thus, according to those studies, more likely to be effective. The discrepancy between the significant number of journals avowing support for research transparency and the relative rarity of existing TOP Level II or TOP Level III journal policies that reflect this support may be due to the perceived notion that the additional labor needed to implement such stringent policies is untenable for editorial teams already overburdened with their current manuscript review and publication task lists [14–15]. Currently the Center for Open Science recognizes only 16 journals as satisfying Top Level III by using shared data, code, and materials to reproduce computational findings in manuscript submissions prior to publication [16].

This phenomenon suggests the need to consider the effectiveness of journal data policies to present and articulate transparency requirements, and how well they facilitate (or hinder) authors’ ability to produce and provide access to data, code, and associated materials that meet quality standards for computational reproducibility.

With that objective in mind, we conducted a mixed-methods study that examined individual data policies alongside editors’ and authors’ interpretation of policy requirements to answer the following research questions:

1. Do journal-issued data policies articulate clear requirements for data, analytic methods, and research materials sharing and/or verification of computational reproducibility as prescribed in TOP Guidelines? Do editors and authors understand journal-issued data policy requirements as stated in the policy language?
2. Do journal-issued data policies use language that obliges authors to satisfy data policy requirements?

If the scientific community is to actualize the principles of openness, transparency, and reproducibility the TOP Guidelines are meant to uphold, it is critical that researchers understand the requirements of journal data policies so as to satisfy those requirements. In this paper, we describe the first phase of a larger study to develop a model for robust data policy implementation that supports transparent research practices. Based on our initial findings, we present recommendations on how to present data policies to authors in a way that most effectively articulates editors’ expectations and elicits authors’ clear understanding of the actions necessary for them to fulfill those expectations.

## Materials and methods

Human subject research was approved by the University of North Carolina at Chapel Hill Institutional Review Board (IRB#18–0295, 17–2143). Electronic consent was obtained from study participants via online survey instrument.

### Editor survey

The editor survey was designed to identify journals that have a TOP Level II or TOP Level III policy in place and, for those particular journals, to collect data on how these policies are communicated to authors and reviewers; whether or not the policy is enforced and by what means; and challenges and strategies of successful policy implementation. The survey instrument included a combination of multiple choice, Likert scale, and open-ended questions, with programmed skip patterns enabled to allow for omission of question items not applicable to certain respondents (see **S1 Appendix**). The survey was administered using the Qualtrics web survey platform.

The editor survey sample was generated by selecting the top 250 ranked journals in the biological, health, and social sciences subject domain categories according to their Eigenfactor scores published in the 2016 InCites Journal Citation Report [17]. Inclusion of titles in the three specified subject domains required selection of InCites categories that corresponded with the three broader study-specified domains. In some cases, journal titles appeared in more than one category. For these journals, we made a determination of which domain the journal should be placed based on a closer examination of the disciplinary focus of the journal.

An invitation to participate in the survey was sent via email in December 2017 to 702 journal editors for whom we were able to locate email addresses from publicly available web resources. Delivery of 18 of the 702 emails failed (i.e., “bounced”). When also taking into account direct email replies from 4 individuals who indicated that they were no longer serving as journal editor and 2 cases in which the email address was no longer valid for the intended recipient, there was a total of 678 potential survey respondents. Approximately two weeks after delivery of the initial invitation, a reminder email was sent to individuals who had not completed the survey, with a second reminder sent to non-respondents approximately two weeks after that. 113 respondents participated in the survey to yield a 16.7% response rate.

### Author survey

The author survey instrument included questions that helped to determine the degree of individual authors’ awareness and understanding of the data policy of the journal in which they published. Additional questions asked about authors’ challenges while performing specific tasks to fulfill data policy requirements. Authors who have also served as peer reviewers were presented with additional questions related to their experiences with data policy implementation from a peer reviewer’s perspective. These questions focused on their process and challenges of evaluating underlying data, code, and/or associated research materials.

The survey instrument included a combination of multiple choice, Likert scale, and open-ended questions, with programmed skip patterns to allow the survey to omit questions not applicable to individual respondents (see **S2 Appendix**). The survey was administered April to May 2018 using the Qualtrics web survey platform.

Based on the editors’ survey responses, we identified the journals from our list with a data policy that: 1) required authors to provide or describe access to data, code, and/or other research materials associated with the article and/or 2) included a policy enforcement mechanism. This yielded a list of 51 journals. We then examined the first 10 research articles in the current issue of each journal and compiled a list of the corresponding authors. If a journal did not designate a corresponding author, we used the first author. If an issue did not contain 10 research articles, we obtained authors from the preceding issue. Editorials, reviews, letters, and other types of articles not reporting empirical results were omitted. This exercise yielded 510 authors for our author survey. Using publicly available web resources, we identified email addresses for all 510.

Email invitations to participate in the survey were sent to the 510 authors in April 2018. Eight bounced and in one case the author was on sabbatical and unable to reply. Of the resulting 501 potential respondents, 80 participated in the survey, yielding a 16.0% response rate. The 80 respondents represented 34 of the 51 data policy-issuing journals we identified in the editor survey.

### Policy content analysis

Data policies were analyzed for journals for which the editor responded “Yes” to the question, “Has [Journal] issued a policy that requires authors to provide access to data, code, and/or other research materials underlying research findings presented in their articles?” and confirming that the policy required the authors to do any one of the following:

- Submit data underlying article findings to a trusted repository
- Submit analytic methods (e.g., code, scripts, packages) to a trusted repository
- Submit research materials (e.g., codebook, readme files) to a trusted repository
- Explain access restrictions for data that cannot be shared due to legal or ethical reasons
- Describe the process for accessing data that cannot be shared due to legal or ethical reasons
- Other (Please specify)

Editors who selected the “Other” category most often specified policy details that echoed that of the predefined categories including policy exemptions for legal and ethical reasons, and provisions for repository deposition of data.

Journals that did not meet the above criteria, but for which the editor did specify a mechanism for data policy enforcement (“Which of the following procedures are included in data policy compliance checks to ensure authors have fulfilled policy requirements?”) were also included in the sample for policy analysis.

For each of the selected journals, we located language text on each journal’s website that referenced instructions to authors, editorial policies, data policies, and/or any other text that provided information to authors on specific requirements for manuscript submission and publication. Editors were also given the opportunity in the survey to provide a URL to the policy or upload policy documents. If relevant language was not located on a journal’s primary website or submitted by the editor, we attempted to locate policy information within the front-facing pages of the manuscript submission portal.

Available policy text was imported into the MAXQDA qualitative data analysis software platform to apply a coding scheme to indicate the presence of a data policy and, if present, any articulation of requirements for data, analytic methods (code), and/or research materials transparency in accordance with the strength of language and conditions as stipulated by TOP Guidelines. Codes were applied to text that indicated specific requirements (i.e., availability statement, repository deposit) as well as mechanisms for policy enforcement (i.e., verification of computational reproducibility).

The content analysis was performed independently by two project team members and tested for intercoder agreement. Each coder reviewed the policy text and identified terms that specified the policy conditions, strength of language, and relevant terms used to describe the type of transparency requirement (i.e., analytics methods (code), data, research materials). In cases for which there were differences in coding, team members reexamined the policy to reach consensus on the most appropriate code application in accordance with the established code definitions.

Data files for the editor survey, author survey, and policy content analysis are available in the UNC Dataverse Repository at https://doi.org/10.15139/S3/DKOUDY [18].

## Results

### Data policies according to editors

Of the 113 editors who responded to the survey, 57.5% (n = 65) reported their journal having a data policy (see S1 Table). However, when these editors were asked specifically about policy content, only 45.1% (n = 51) specified the presence of transparency requirements. Of these 51 editors, 66.7% (n = 34) indicated a data transparency requirement, 54.9% (n = 28) indicated an analytic methods (or code) transparency requirement, and 37.3% (n = 19) indicated a research materials transparency requirement. S2 Table shows the breakdown by domain of journals and policy requirements as reported by editors.

TOP Level III policies that included provisions for verifying computational reproducibility of reported results were rare, with only 11.8% (n = 6) of editors reporting verification of findings using authors’ submitted code, data, and associated research materials as being part of the policy (S3 Table).

In their open-ended responses to the question, “*What have been the greatest challenges of data policy implementation for [the journal]*?”, more than one editor mentioned policy enforcement. One respondent provided details: “I'm quite sure that some of the data sets we've accepted are not in very good shape, and editors and reviewers don't always take the time to check. Fully implementing this would require additional staff and funding and cannot be done by volunteer editors and reviewers.”

When it comes to policy implementation success, respondents mentioned both social and technical aspects as contributors to success. More than one noted the advantages of community consensus and engagement in the implementation of data policies. In an open-ended survey question, “*What strategies or mechanisms have contributed most to the success of data policy implementation for [the journal]*?”, respondents cited “Developing policies with significant input from stakeholders and broad consensus,” “Strong community buy-in,” and “A general acceptance in the community that data should be made available” as exemplifying social contributors to policy success. In terms of technical contributors, some authors made note of their use of data repository services to make the process of submitting data easier for authors.

### Data policies according to authors

Of the 80 authors who responded to the survey, there were 5 (6.3%) who indicated that they were not aware of the data policy at any time during the manuscript review and publication process. For the 73 authors who answered the question, “*How easy or difficult was it for you to understand what was expected of you to fulfill the requirements of the data policy*?”, 86.3% (n = 63) found it somewhat or very easy to understand what was expected of them, whereas 13.7% (n = 10) found it somewhat or very difficult to understand the policy (S4 Table).

Despite the ease with which the majority of authors reported understanding the data policies, a smaller percentage of authors were aware of the specific transparency requirements stated in written policies. For the 34 journals represented in the author survey, 67.6% (n = 23) were noted by at least one author to have a data transparency requirement, 47.1% (n = 16) to have an analytic transparency requirement, and 38.2% (n = 13) to have a research materials transparency requirement written in the policy (S5 Table).

When given the opportunity to share more details of their experience in regard to data policy compliance and/or implementation (*In the space below*, *please share any other information regarding your experience as an author and/or a reviewer complying with or implementing journal data policies*), respondents cited the effort required to prepare data for sharing. The sentiment articulated in the following was shared by more than one author: “Submitting data and code is not ‘challenging’ per se (in the same sense that other aspects of scientific research is challenging), but it does take effort. The level of labeling, commenting, and general tidiness that is acceptable is very different for what a researcher can tolerate in materials for their personal use vs. they want to show others and will be useful for others.”

Like comments from the editor survey, some authors noted the benefit of having a mechanism in place for submitting data to a repository to ease the process of policy compliance: “If data is to be stored elsewhere from the journal (e.g. Dryad) it is much easier when the journal creates the entry and provides the link for upload.”

### Data policy text

The content analysis included the policies for the 51 journals for which editors indicated that a policy is in place. The location of policies on respective journals’ websites varied, with 49.0% (n = 25) embedded within the text of instructions to authors, editorial policies, or similar information describing the manuscript submission process. 33.3% (n = 17) were also embedded within other instructions, but were able to be located by using a link that navigated directly to the data policy section (i.e., indexed). We located other data policies on a dedicated webpage on the journal website (5.9%; n = 3) and in downloadable documents (3.9%; n = 2). We were unsuccessful in locating the data policy language for 7.8% (n = 4) of journals. S6 Table provides a breakdown of policy locations by domain.

Of the 47 policies we were able to locate, 100.0% included language that mentions data transparency, 73.7% (n = 38) included mention of analytic methods transparency, and 70.2% (n = 33) mentioned research materials transparency (S7 Table).

While the majority of journal policies include mentions of transparency requirements, it was not a straightforward process to determine the stringency, or strictness, of the policy. In many cases, policy language was vague or contradicted itself with expressions of both encouragement and requirement in different parts of the policy text. This experience is consistent with other data policy analysis studies [10, 19–20]. S8 Table lists examples of phrases used to express policy requirements.

We identified 51.1% (n = 24) policies that used terms to convey that transparency was encouraged, and 76.6% (n = 36) that declared transparency a requirement. In 29.8% (n = 14) of the cases, the policy included language indicating both encouragement and requirement of transparency. Just 2 policies from the social sciences included a verification of analysis results requirement (**S9 Table**).

Another confounding aspect of the policies we analyzed was the assortment of terminology used to refer to data, analytic methods, and research materials transparency. For the most part, policies specified “data” or “dataset”, but in several cases, policies referred to data by naming the domain-specific materials used as evidence to support research findings (e.g., “sequences,” “microarrays”). Identifying policies with analytic methods transparency requirements was also straightforward, with use of terms such as “command files,” “scripts,” and “algorithms.” On the other hand, where policies described research materials transparency, the language was vague, referring simply to “materials” or failing to define specific types of materials (e.g., “documentation,” “metadata,” “other artifacts).” S10 Table provides examples of terminology we interpreted to refer to data, analytic methods, and research materials.

### Editor, author, and policy alignment

**Fig 1:**
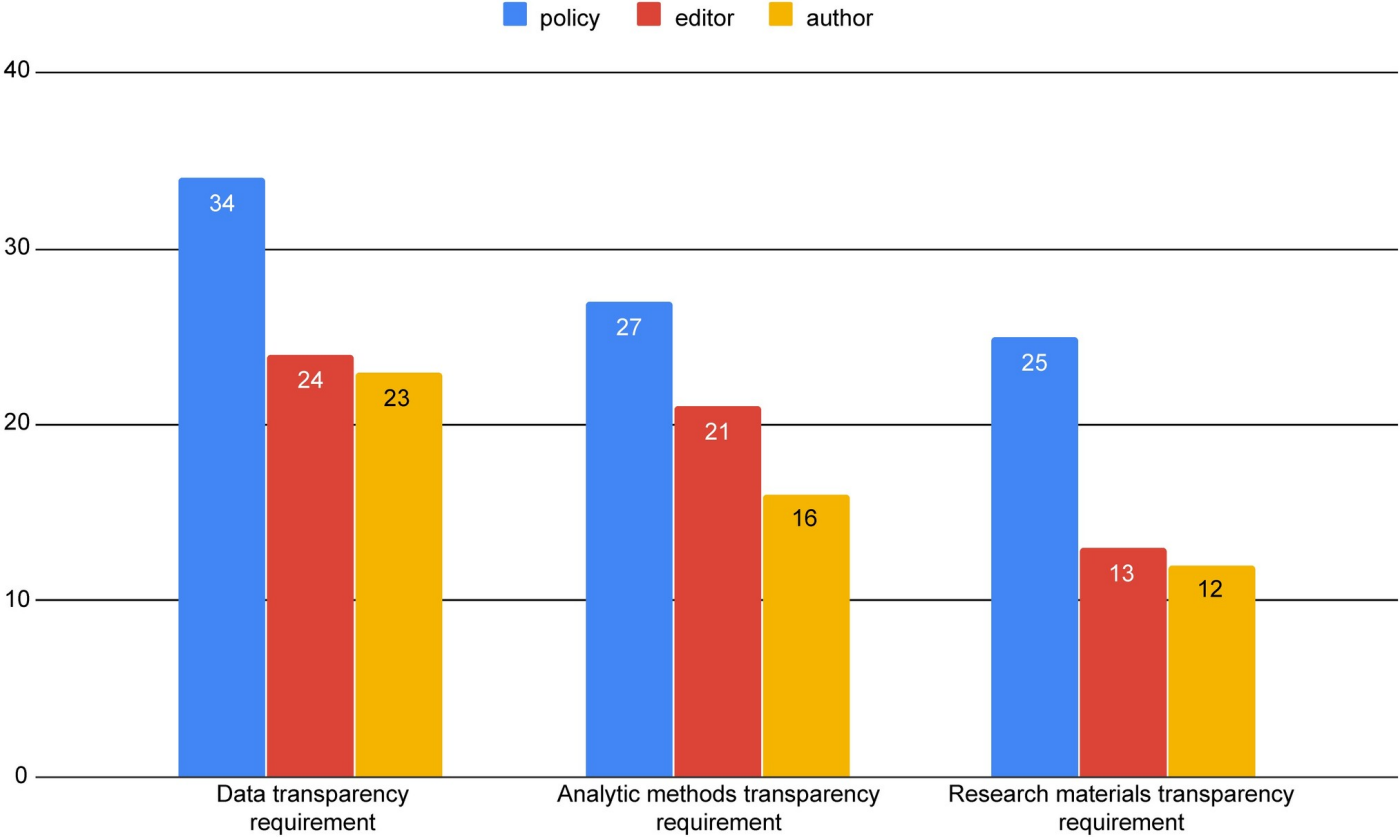
Comparison of editors’ conception of the policy, authors’ understanding of the policy, and the policy as written.

When we review the results of the surveys and policy content analysis in parallel, we can begin to answer the research questions we sought to address. Comparing editors’ survey responses, authors’ survey responses, and the policy content (where a journal is represented in both the editor and author surveys), we discovered discrepancies between what editors declare their policies to require, what authors understand the policy to require, and what the policy itself states as requirements (see Fig 1). For all categories of transparency requirements, editors underestimate their own journals’ policies. Likewise, authors underestimate policy requirements, but to a greater degree. Even though all 34 policies in the comparison mention data transparency, only 24 editors and 23 authors identified this requirement. For the 27 policies that included an analytic transparency requirement, this was recognized by only 16 authors, and 21 editors. For the research materials transparency requirement, 13 editors and 12 authors were closer in agreement with its presence but is about half the number of the 26 policies that did state this requirement.

**Fig 2:**
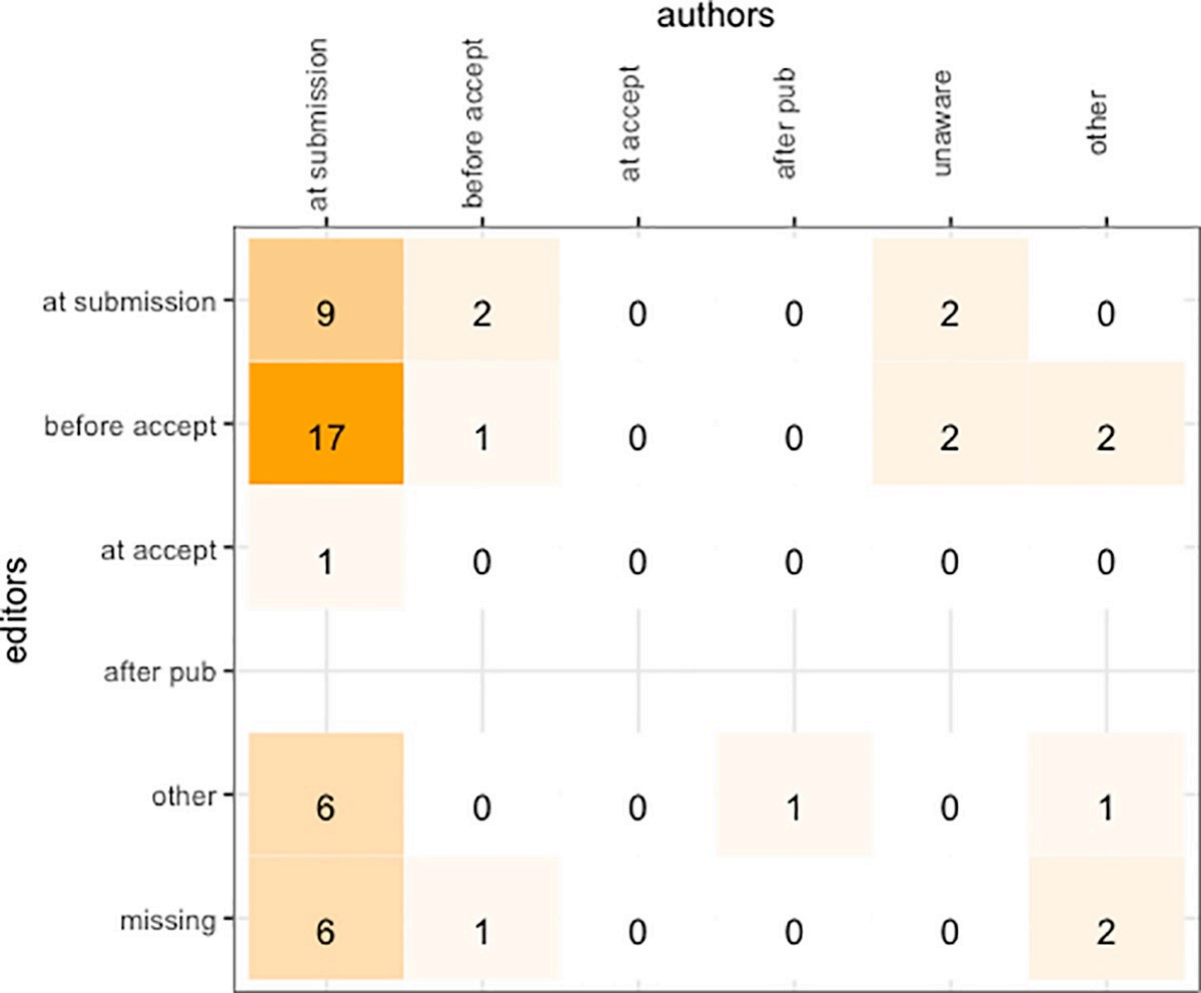
Comparison of editors’ and authors’ responses regarding the timing at which journal data policy guidance is communicated to authors.

There was also a discrepancy between editors and authors as to their understanding of when the journal policy’s data guidance was presented to authors (Fig 2). When asked “At what point during the manuscript review and publication process did you become aware of the [journal’s] data policy requirements?”, 74% (39/53) of the authors responded, “At the time of initial manuscript submission”. The editors of the same journals as those 39 responses gave a congruent response only 23% (9/39) of the time. Almost half (46%, or 18/39) of the editors responded that the data policy was communicated after peer review, either before or upon final manuscript acceptance, but only *one* of the 53 authors responded that the data policy was communicated after peer review. While not all the responses can be mapped between the author and editor surveys, only 10 of the 53 (19%) responses strictly agree. It is important to emphasize that this is disagreement between the editor and authors *of each individual journal*. In the five cases where the author selected “Other”, all but one of the free text responses indicate that the author already knew the data policy prior to selecting the journal, and the remaining author could not recall the timing of when they learned about the data policy. Interestingly, four of the authors responded that “I was not aware of the data policy at any point during the manuscript submission and publication process”.

## Discussion

A limitation of the study was the response rates for both the editor and author surveys, which precluded a balanced investigation into the three disciplinary domains we sought to study. While the response rates were comparable to similar studies, the low response rates may be partly attributed to a number of factors including the delivery and content of the survey instrument. Web-based surveys tend to yield lower response rates than other modes of delivery, and participation is less likely when the topic is not salient to potential respondents [21]. Despite our intention to study journals across the biological, health, and social sciences, health sciences journals were vastly underrepresented in the study. It is understood that data sharing is not as widespread in the health sciences as in other domains due to ethical concerns with human subjects research [22–24]. This disposition among the health sciences community may have rendered the survey topic unimportant to individuals associated with this domain. With so few participants representing the health sciences, the results we report rely heavily on survey responses from journal editors and authors from the biological and social sciences and limit our understanding of the complexities of data policy implementation within the health sciences community.

Aside from disciplinary differences, there are additional factors that may explain the reasons for the misalignment among editors’ conception of the policy, authors’ interpretation of the policy, and the policy itself. When identifying journals’ data policies, we found that some journals presented their data policy as a section in the author instructions, while others dedicated an entire webpage to outline data policy requirements and instructions. The prominence and comprehensiveness of the policy also may have an impact on the effectiveness of the policy to communicate its intended message. In addition to the location of policy text on the journal website, some journal policies included value statements that justified the journal’s adoption of the policy. Variations of statements declaring the journal’s commitment to open science, support for maximum reuse of research materials for scientific advancement, and promotion of scientific integrity through verification of published results were some of the sentiments included with data policies. Whether or not inclusion of this information enhances understanding of and compliance with the policy is also worthy of further investigation.

By comparing journal-issued data policy content with the editors’ and authors’ understanding of the policy requirements, our research adds an important dimension to the recently published studies that place primary focus on the policy artifact. While some of these studies considered contextual factors such as the journal’s impact factor, publisher, professional society affiliation, and open access status to assess the effectiveness of the policy in promoting data sharing practices [10, 13, 25–26], our study adds the critical perspective of the very individuals who issue and enforce these policies, as well as those who are required to comply with the policies.

Whether or not policies effectively promote research transparency by mandating data sharing depends firstly on whether or not these policies effectively communicate data sharing requirements and obligate researchers to satisfy the requirements. The lack of agreement about the content of policies among editors, authors, and the policies themselves suggests a significant breakdown in communication that is likely to hinder compliance, which in turn affects enforcement. These findings support the following answers to our research questions:

### Do journal-issued data policies articulate clear requirements for data, analytic methods, and research materials sharing and/or verification of computational reproducibility as prescribed in TOP Guidelines?

All of the data policies we examined included statements describing data transparency, and the majority described analytic methods and research materials transparency. However, it was less clear whether the intention of the policy was to encourage or require transparency practices. In some cases, the policy contradicted itself with the appearance of phrasing that suggested both encouragement and requirement of a particular transparency practice. Because of this ambiguity, many policies did not fit neatly, if at all, into the TOP Guidelines matrix to differentiate among TOP levels of stringency. If policies intended to reflect standards of transparency and rigor, this intention was not manifested consistently in policy language.

### Do editors understand the journal-issued data policies as they have been written?

A number of editors who responded to the survey understated the requirements as written in the policy documents. All correctly confirmed the presence of a data policy for their journals; however, some editors did not recognize the presence of additional transparency requirements for analytic methods or research materials. Others misstated whether transparency was encouraged or required. This discrepancy suggests that editors are not fully knowledgeable of how policies may be (mis)interpreted by authors.

### Do journal-issued data policies use language that obligates authors to comply with the policy?

To an even greater degree than editors, authors are unaware of the specific transparency requirements stated in the data policy language. Nonetheless, authors are well aware of the effort involved in sharing data. For authors to feel obligated to comply with a journal-issued data policy, they need to know of the conditions set forth in the policies to compel them to invest the effort to prepare materials for sharing. Our findings suggest that data policies do not effectively communicate the journal’s expectations in a way that compels authors to fulfill those expectations.

### Recommendations

For journals considering implementing a data policy—and those seeking greater policy compliance—it is important that policies are clearly articulated and accurately understood. Here, we offer four specific recommendations. While the extent to which following these recommendations will contribute to a shared understanding of the data policy among editors and authors cannot easily be predicted, they are relatively straightforward to implement and are under the journal’s control.

#### Recommendation 1. Engage the stakeholder community in the development and implementation of data policies

Survey results showed that journals citing successful data policy compliance credited community consensus as well as community engagement as positive factors. Editors mentioned fostering discussions in conference workshops and professional meetings to achieve broad consensus and aligning policy motivations with the same assumptions that have bolstered acceptance of the imperative to share data as strategies contributing to the success of their policies. These types of stakeholder engagement offer important opportunities for editors and authors to establish a shared understanding of policy expectations, which in turn enables authors to better anticipate and understand data policy requirements.

Based on editors’ reported experiences in establishing data policies, we recommend that editors make it a point to involve authors in the development of policies that use language mutually understood and to describe expectations that are mutually accepted.

#### Recommendation 2. Express data policy requirements with clear and consistent language

Journals wishing to implement a data review policy should be explicit in their expectations and use language that is precise and unambiguous to members of the community it represents. Ambiguous policy language has made it difficult for authors to understand a policy’s requirements or procedures [10, 25]. This is especially likely when there is ambiguity in whether or not depositing data, code, and related materials to a repository is required or encouraged by the journal, as seen in S9 Table in which almost a third of policies studied do both. For some of the data policies evaluated, particularly when embedded in various sections of exhaustive author instructions, statements of “requirement” conflicted with subsequent explanations of “recommended” transparency practices. One source of confusion might be different expectations for different kinds of data (e.g. a requirement for submission of DNA data to GenBank but no such requirement for other data), when such distinctions have not been clearly stated.

In addition to using declarative statements on policy requirements, these requirements should be described in a way that specifies the parameters of conditions for compliance. The various ways the policies we studied referred to “data, “analytic methods,” and “research materials,” as seen in S10 Table likely contribute to the misunderstanding of the policies’ intended requirements. Referring simply to “data” when stating a data sharing requirement, for example, gives little instruction to authors as to whether authors must share raw data, analysis data, or other type of evidence necessary to verify results—particularly for specialized disciplines in which such distinctions are blurred. Requiring authors to share materials without specifying acceptable platforms for sharing data may result in the data becoming inaccessible if not placed in a trusted repository. Without a definition of “research materials,” authors may have little reference as to what this refers and not make available the materials needed for the community to reproduce results.

We recommend that journal policy language be resolute in what is required of authors to comply with the policy and enable compliance by providing prescriptive definitions of terms used to describe expectations for policy compliance.

#### Recommendation 3. Align policy requirements with standards and best practices

Following the previous recommendation, journal-issued data policies are a point of discussion within a larger, ongoing conversation about scientific reproducibility. Open science advocates, data repositories, scholarly publishers, academic societies, and other stakeholders in the scientific enterprise have begun to converge on the principles and practices that support transparent research, which currently are not presented consistently across data policies [20]. Many of the requirements imposed by the policies we studied included a data sharing requirement alone, which is insufficient without accompanying code and other related materials to yield reproducible research. A common understanding of the expectation for sharing data, code, and materials to allow for verification of published results is an important aspect of data policy implementation if intended to increase transparency and reproducibility. Fortunately, community-approved expectations have been codified in standard frameworks that outline prerequisites for reproducible research and strategies for satisfying them by experts who are versed in data management standards as well as research practice norms. These standards include best practices for anonymization and other protections for sensitive data where transparency is potentially unethical.

The TOP Guidelines in particular were created by an interdisciplinary committee of critical stakeholders to “translate scientific norms and values into concrete actions” through standards of scientific transparency upheld through journal policy implementation [27] and have over almost 5,000 journal signatories. We recommend that journals craft data policies that use language to stipulate conditions and levels of enforcement as prescribed by prevailing standards such as the TOP Guidelines and with respect to the particular norms of the community. Considered together with the specific norms and issues within the specific research communities [28], policy language will be more effective in aligning principles with practice.

#### Recommendation 4. Collaborate with data repository experts for data policy implementation support

Based on the content analysis of existing data policies, it is evident that policies were not written with data curation, management, and preservation standards in mind. For the scientific community to reap the benefits of data access and research transparency, researchers must follow data management best practices for producing high-quality data packages for subsequent submission to a trusted data repository. Because data management has not yet been identified as essential training for scientists [29–31], it stands to reason that many editors and authors are not versed in the necessary tasks to ensure data are accessible, interpretable, and usable. Furthermore, earnest attempts to execute these tasks (or confirm that these tasks were performed in accordance with policy) take time and effort that researchers are not eager to expend.

This does not have to be the case. As noted by authors in their survey responses, data repositories have tools and services to help alleviate the additional burden that data policy mandates place on editors and authors [32–33]. Repository staff are able to recommend policy language that specifies the actions necessary for effective data sharing, provide repository support for data submissions, develop guidance documents that outline and define policy requirements, and provide user support services for packaging and sharing data.

Therefore, we recommend that journals establish relationships with data repositories to ensure that policies include standards-based requirements and specify the infrastructure to use for fulfilling requirements.

## Conclusion

Scholarly journals are aware of the potential they have to effect change in normative research practice to one that includes research transparency. By issuing data policies that make article publication contingent on authors producing and sharing quality data, code, and other research materials supporting their reported findings, researchers have little choice but to comply if they are to produce the publication outputs on which academic tenure and promotion structures place significant weight [34–35]. This logic assumes that journal-issued data policies will obligate researchers to engage in specific activities to ensure that the research materials underlying published materials are discoverable, understandable, and reusable.

For this assumption to hold, however, policies must be communicated in a way that enhances the authors’ understanding of the journals’ expectations and the specific processes for meeting those expectations. Based on our analysis of existing journal-issued data policies alongside survey responses from authors and editors in regard to those policies, this is not always the case. Many of the policies we examined used vague, non-standard language, failed to make definitive statements as to what materials were required, and offered little guidance as to how to achieve compliance. Additionally, we find a discrepancy between when authors are looking for the data policy (generally, prior to article submission) and when editors expect to communicate it to authors (often after peer review). As a result, the data policy itself has the potential to be “lost in translation”. We offer a number of recommendations to journals that are relatively straightforward to implement and which we would hypothesize to alleviate some of the discrepancies noted here, although understanding their effectiveness will require additional study.

The next steps of this study are to synthesize these findings into a TOP Level III data policy model that offers standardized language to articulate policy requirements as well as guidance for editors and authors on policy implementation and compliance, respectively. This policy model will be informed by the findings of the current study as well as qualitative data from in-depth interviews, to be reported separately, that aimed to better understand the experiences of editors and reviewers implementing data review policies. In doing so, editors—along with members of their stakeholder community—will have the benefit of evidence-based guidance to support the development of an effective data policy that promotes research transparency as part of normative research practice.

## Supporting information

S1 Appendix Editor survey instrument. (PDF)

S2 Appendix Author survey instrument. (PDF)

S3 Appendix List of journals included in study. (PDF)

S1 Table Editor indication of presence of data policy. (DOCX)

S2 Table Editor indication of specific transparency requirements. (DOCX)

S3 Table Editor indication of reproducibility verification requirement. (DOCX)

S4 Table Authors’ reported ease or difficulty in understanding journal-issued data policy requirements. (DOCX)

S5 Table Author understanding of policy journal-issued data policy requirements. (DOCX)

S6 Table Location of data policy text. (DOCX)

S7 Table Mention of transparency requirements in policy language. (DOCX)

S8 Table Excerpts from policy text used to establish encouragement or requirement of policy conditions. (DOCX)

S9 Table Stringency of transparency requirements as expressed in policy language. (DOCX)

S10 Table Examples of terms used in policies to refer to categories of transparency requirements. (DOCX)
