# Supplementary material for: Journal data policies: Exploring how the understanding of editors and authors corresponds to the policies themselves
Source: PLoS One. 2020 Mar 25;15(3):e0230281. doi: 10.1371/journal.pone.0230281 (PMC7094825; doi:10.1371/journal.pone.0230281)
Supplement: S1 Appendix — (PDF) [file pone.0230281.s001.pdf]

RWJF_EditorSurvey

Start of Block: Default Question Block

INTRO Thank you for participating in the Journal Editors' Survey on Data Policy Implementation. The information you provide will help identify the most effective and efficient methods for implementing robust data policies. This survey is being conducted by the Odum Institute at the University of North Carolina at Chapel Hill as part of a larger research study funded by the Robert Wood Johnson Foundation (#OAR 74419). This study aims to develop an evidence-based model for data policy implementation that yields the greatest degree of access to quality research data.   Your participation is voluntary; you may end the survey at any time. You will NOT be individually identified in any reports or studies that are produced; only aggregate results will be reported. The survey will take approximately 15 minutes to complete.   If you have any questions or concerns about the study, please contact the project team at odumarchive@unc.edu or 919-962-6293.   **Please click on the arrow [>>] button below to begin the survey.**

Page Break

QPOL Has *${m://ExternalDataReference}* issued a policy that requires authors to provide access to data, code, and/or other research materials underlying research findings presented in their articles?

- O Yes (1)
- O No (3)

Skip To: End of Survey If QPOL = No

Page Break

Q1 What proportion of articles published in *${m://ExternalDataReference}* report findings from research studies based on empirical data?

- O Less than 50% (1)
- O More than 50% (2)
- O ${m://ExternalDataReference} does not publish empirical research studies. (3)

Skip To: End of Survey If Q1 = ${m://ExternalDataReference} does not publish empirical research studies.

Page Break

Q2 Does the data policy issued by the *${m://ExternalDataReference}* require authors to do the following?

	Yes (1)	No (2)
Submit data underlying article findings to a trusted repository (1)	o	o
Submit analytic methods (e.g., code, scripts, packages) to a trusted repository (2)	o	o
Submit research materials (e.g., codebook, readme file) to a trusted repository (3)	o	o
Explain access restrictions for data that cannot be shared due to legal or ethical reasons (4)	o	o
Describe the process for accessing data that cannot be shared due to legal or ethical reasons (5)	o	o
Other (Please specify): (6)	o	o

Page Break

Q3 Does implementation of the *${m://ExternalDataReference}* data policy include the following provisions?

	Yes (1)	No (2)
Policy exemptions for data that cannot be shared for legal or ethical reasons (2)	o	o
Sanctions for failure to comply with the data policy (3)	o	o

Page Break

*

Q4 When was the current *${m://ExternalDataReference}* data policy issued?
Please enter MM/DD/YYYY.

____

Page Break

Q5 At what point during the manuscript publication process are authors required to submit their data, code, and/or other associated materials?

- O At the time of manuscript submission (1)
- O After manuscript peer review, but prior to final manuscript acceptance (i.e., final manuscript acceptance is contingent on submission of data, code, and/or associated research materials) (2)
- O Upon final manuscript acceptance (3)
- O After article publication (4)
- O Other (Please specify): (5) ____

Page Break

Q6 Does *${m://ExternalDataReference}* provide additional instructions, guidance documents, or other information to assist authors with data policy compliance that are not publicly accessible on the journal website?

- O Yes (1)
- O No (2)

Q6b If additional data policy instructions/guidance documents for **authors** are not publicly accessible online, please upload them here. For multiple files, please combine them into a single .zip file.

Page Break

Q7 At what point during the manuscript publication process are the additional data policy instructions/guidance documents provided to authors?
Select all that apply.

- ▢ At the time of manuscript submission (1)
- ▢ After manuscript peer review, but prior to final manuscript acceptance (i.e., final manuscript acceptance is contingent on submission of data, code, and/or associated research materials) (2)
- ▢ Upon final manuscript acceptance (3)
- ▢ After article publication (4)
- ▢ Other (Please specify): (5) ____

Page Break

Q8 What are common questions or feedback you have received from **authors** regarding the data policy?

____

____

____

____

____

Page Break

Q9 Which of the following procedures are included in data policy compliance checks to ensure authors have fulfilled policy requirements?
Select all that apply.

- ▢ ⊗Procedures are not in place to ensure policy compliance. (6)
- ▢ Review of manuscript for presence of a data citation for the author's data, code, and/or associated materials (1)
- ▢ Confirmation of the presence of author's data, code, and/or associated materials at the location indicated by the data citation (2)
- ▢ Examination of the author's data, code, and/or associated materials for quality and/or completeness (3)
- ▢ Verification of findings using author's submitted data, code, and/or associated research materials to ensure reproducibilty of published results (4)
- ▢ Other (Please specify): (5) ____

Page Break

Carry Forward Selected Choices from "Q9"

Q10 Who is responsible for performing each of the following data compliance check procedures?

	Editor (1)	Peer reviewer(s) (2)	Other journal staff (3)	Third party (4)
⊗Procedures are not in place to ensure policy compliance. (x6)	o	o	o	o
Review of manuscript for presence of a data citation for the author's data, code, and/or associated materials (x1)	o	o	o	o
Confirmation of the presence of author's data, code, and/or associated materials at the location indicated by the data citation (x2)	o	o	o	o
Examination of the author's data, code, and/or associated materials for quality and/or completeness (x3)	o	o	o	o
Verification of findings using author's submitted data, code, and/or associated research materials to ensure reproducibilty of published results	o	o	o	o

(x4)				
Other (Please specify): (x5)	o	o	o	o

Page Break

Display This Question:

If Q10 = Peer reviewer(s)

Q11 Does *${m://ExternalDataReference}* provide additional instructions, guidance documents, or other information to assist **peer reviewers** with data policy implementation that are not publicly accessible on the journal website?

O Yes (1)

O No (2)

Display This Question:

If Q10 = Peer reviewer(s)

Q11b If the additional data policy instructions/guidance documents for **peer reviewers** are not publicly accessible online, please upload them here. For multiple files, please combine them into a single .zip file.

Page Break

Q12 What are common questions or feedback you have received from **peer reviewers** regarding the data policy?

____

____

____

____

____

Page Break

Q13 Does *${m://ExternalDataReference}* offer any tools, services, and/or any other provisions to enable authors to submit data they would otherwise not be able to share due to legal or ethical reasons, for purposes of data policy compliance?

- O Yes (1)
- O No (2)

Page Break

Display This Question:

If Q13 = Yes

Q13a Please describe the tools, services, and/or other provisions *${m://ExternalDataReference}* offers that enable researchers to submit data they otherwise would not be able to share due to legal or ethical reasons, for purposes of data policy compliance.

____

____

____

____

____

Page Break

Q14 What have been the greatest challenges of data policy implementation for *${m://ExternalDataReference}*?

____

____

____

____

____

Page Break

Q15 What strategies or mechanisms have contributed most to the success of data policy implementation for *${m://ExternalDataReference}*?

____

____

____

____

____

Page Break

Q16 In the space below, please share any other information that will help us to understand the implementation of the *${m://ExternalDataReference}* data policy.

____

____

____

____

____

Page Break

END **You have reached the end of the survey**.
By clicking on the advance button [>>] below, your survey will be finalized, and you will NOT be able to return to the survey to review or edit your responses.
If you would like to review or edit your responses at a later time, *please close your browser window now*. You can return to your saved survey at any time using the survey link you received in the email invitation.

End of Block: Default Question Block
