# Supplementary material for: Journal data policies: Exploring how the understanding of editors and authors corresponds to the policies themselves
Source: PLoS One. 2020 Mar 25;15(3):e0230281. doi: 10.1371/journal.pone.0230281 (PMC7094825; doi:10.1371/journal.pone.0230281)
Supplement: S2 Appendix — (PDF) [file pone.0230281.s002.pdf]

RWJF_AuthorSurvey

Start of Block: Default Question Block

INTRO Thank you for participating in the Authors' Survey on Journal Data Policy Implementation. You were selected to participate in this study because you authored an article in a journal that has a data policy in place that may have required you to make your data available as part of the manuscript review and publication process. The information you provide will help identify the most effective and efficient methods for implementing robust data policies. This research survey is being conducted by the Odum Institute at the University of North Carolina at Chapel Hill as part of a larger study funded by the Robert Wood Johnson Foundation (#OAR 74419).

Your participation is voluntary and there is no compensation for participating in this research; you may skip any question you do not want to answer. You will NOT be individually identified in any reports or studies that are produced. The survey will take approximately 10 minutes to complete. While measures have been put in place to prevent confidentiality breaches, there is a chance that your information may be accidentally disclosed.

If you have any questions or concerns, please contact Thu-Mai Christian, principal investigator, at thumai@email.unc.edu or 919-962-6293.

By clicking on the arrow button below to being the survey, you are consenting to participate in this research study.

Page Break

REF1 **This first set of questions will ask about your experience as an author submitting a manuscript for publication in *${e://Field/Journal}*, and that was published in the ${e://Field/Month} ${e://Field/Year} issue. When answering these questions, please refer to this particular experience.**

Q1 At what point during the manuscript review and publication process did you become aware of the *${e://Field/Journal}* data policy requirements?

- O At the time of initial manuscript submission (1)
- O During notification of revise and resubmit (2)
- O After manuscript peer review, but prior to final manuscript acceptance (3)
- O Upon final manuscript acceptance (4)
- O After article publication (5)
- O Other (Please specify): (6) ____________________
- O I was not aware of the data policy at any point during the manuscript submission and publication process. (7)

Skip To: REF2 If Q1 = I was not aware of the data policy at any point during the manuscript submission and publication process.

Page Break

Q2 Did the data policy issued by *${e://Field/Journal}* require you to do the following?

	Yes (1)	No (2)
Submit data underlying article findings to a trusted repository (1)	o	o
Submit analytic methods (e.g., code, scripts, packages) to a trusted repository (2)	o	o
Submit research materials (e.g., codebook, readme file) to a trusted repository (3)	o	o
Explain access restrictions for data that cannot be shared due to legal or ethical reasons (4)	o	o
Describe the process for accessing data that cannot be shared due to legal or ethical reasons (5)	o	o
Other (Please specify): (6)	o	o

Page Break

Carry Forward Selected Choices from "Q2"

X→

Q3 How challenging did you find the following aspects of fulfilling data policy requirements?

	Not challenging at all (1)	Only a little challenging (2)	Somewhat challenging (3)	Very challenging (4)
Submit data underlying article findings to a trusted repository (x1)	o	o	o	o
Submit analytic methods (e.g., code, scripts, packages) to a trusted repository (x2)	o	o	o	o
Submit research materials (e.g., codebook, readme file) to a trusted repository (x3)	o	o	o	o
Explain access restrictions for data that cannot be shared due to legal or ethical reasons (x4)	o	o	o	o
Describe the process for accessing data that cannot be shared due to legal or ethical reasons (x5)	o	o	o	o
Other (Please specify): (x6)	o	o	o	o

Page Break

Q4 How easy or difficult was it for you to locate information regarding the data policy?

- O Very difficult (1)
- O Somewhat difficult (2)
- O Somewhat easy (3)
- O Very easy (4)

Page Break

Q5 How easy or difficult was it for you to understand what was expected of you to fulfill the requirements of the data policy?

- O Very difficult (1)
- O Somewhat difficult (2)
- O Somewhat easy (3)
- O Very easy (4)

Page Break

Q6 Did *${e://Field/Journal}* provide you with any instructions or guidance documents to assist you in fulfilling the policy requirements in addition to the data policy language?

- O Yes (1)
- O No (2)

Page Break

REF2 **The next few questions will ask about your experience as a peer reviewer.**

Q7 Have you served as a peer reviewer for *${e://Field/Journal}* since the data policy took effect?

- O Yes (1)
- O No (2)

Skip To: Q9 If Q7 = Yes

Page Break

Q8 Have you ever served as a peer reviewer for a journal that has a data policy requiring authors to submit data, code, and/or associated research materials associated with their articles?

- O Yes (1)
- O No (2)

Skip To: Q13 If Q8 = No

Page Break

Q9 As a peer reviewer, have you ever done any of the following as part of your review process?

	Yes (1)	No (2)
Requested access to authors' data, code, and/or associated research materials (1)	o	o
Obtained authors' data, code, and/or associated research materials (2)	o	o
Evaluated authors' data, code, and/or associated research materials (3)	o	o

Skip To: Q12 If Q9 = Obtained authors' data, code, and/or associated research materials

Page Break

Q10 Did you apply the following criteria in your evaluation of authors' data, code, and/or research materials associated with a manuscript under your review?

	Yes (1)	No (2)
Data, code, and/or associated research materials were cited in the manuscript (1)	o	o
Data, code, and/or associated research materials could be accessed from a trusted repository (2)	o	o
Data, code, and/or associated research materials included documentation sufficient to enable interpretation and verification of results (3)	o	o
Data, code, and/or associated research materials could be used to reproduce results presented in the manuscript (4)	o	o
Other (Please specify): (5)	o	o

Page Break

Carry Forward Selected Choices from "Q10"

X→

Q11 How challenging was it to evaluate the authors' data, code, and/or associated research materials based on the following criteria?

	Not challenging at all (1)	Only a little challenging (2)	Somewhat challenging (3)	Very challenging (4)
Data, code, and/or associated research materials were cited in the manuscript (x1)	o	o	o	o
Data, code, and/or associated research materials could be accessed from a trusted repository (x2)	o	o	o	o
Data, code, and/or associated research materials included documentation sufficient to enable interpretation and verification of results (x3)	o	o	o	o
Data, code, and/or associated research materials could be used to reproduce results presented in the manuscript (x4)	o	o	o	o
Other (Please specify): (x5)	o	o	o	o

Page Break

Q12 As a reviewer for *${e://Field/Journal}*, were you provided with any instructions or guidance documents to assist you in your evaluation of authors' data, code, and/or associated research materials?

- O Yes (1)
- O No (2)

Page Break

Q13 In the space below, please share any other information regarding your experience as an author and/or a reviewer complying with or implementing journal data policies.

____

____

____

____

____

Page Break

Q17 **You have reached the end of the survey**.
By clicking on the arrow button below, your survey will be finalized, and you will NOT be able to return to the survey to review or edit your responses.
If you would like to review or edit your responses at a later time, *please close your browser window now*. You can return to your saved survey at any time using the survey link you received in the email invitation.

End of Block: Default Question Block
