# Supplementary material for: Journal data policies: Exploring how the understanding of editors and authors corresponds to the policies themselves
Source: PLoS One. 2020 Mar 25;15(3):e0230281. doi: 10.1371/journal.pone.0230281 (PMC7094825; doi:10.1371/journal.pone.0230281)
Supplement: S3 Appendix — (PDF) [file pone.0230281.s003.pdf]

S3 Appendix. List of Journals.

Domain	Journal
biological sciences	ACS Chemical Biology
social sciences	American Economic Journal-Economic Policy
biological sciences	AMERICAN JOURNAL OF HUMAN GENETICS
social sciences	AMERICAN JOURNAL OF POLITICAL SCIENCE
biological sciences	AMERICAN NATURALIST
biological sciences	ANNALS OF BOTANY
biological sciences	Autophagy
health sciences	CIRCULATION
social sciences	COGNITION
biological sciences	ECOLOGICAL APPLICATIONS
biological sciences	ECOLOGY
biological sciences	Ecology and Evolution
social sciences	ECONOMETRICA
biological sciences	eLife
social sciences	ENERGY ECONOMICS
biological sciences	ENERGY POLICY
biological sciences	EVOLUTION
biological sciences	FEMS MICROBIOLOGY ECOLOGY
biological sciences	Frontiers in Plant Science
biological sciences	FUNCTIONAL ECOLOGY
biological sciences	GENETICS IN MEDICINE
biological sciences	Genome Medicine
health sciences	International Journal of Nanomedicine
social sciences	INTERNATIONAL ORGANIZATION
social sciences	INTERNATIONAL STUDIES QUARTERLY
social sciences	JOURNAL OF ARCHAEOLOGICAL SCIENCE
biological sciences	JOURNAL OF BIOGEOGRAPHY

social sciences	JOURNAL OF CONFLICT RESOLUTION
social sciences	JOURNAL OF DEVELOPMENT ECONOMICS
biological sciences	JOURNAL OF ECOLOGY
social sciences	JOURNAL OF EUROPEAN PUBLIC POLICY
biological sciences	JOURNAL OF EVOLUTIONARY BIOLOGY
social sciences	JOURNAL OF EXPERIMENTAL PSYCHOLOGY-GENERAL
biological sciences	Journal of Geophysical Research-Biogeosciences
social sciences	JOURNAL OF HUMAN RESOURCES
social sciences	JOURNAL OF PEACE RESEARCH
biological sciences	LIMNOLOGY AND OCEANOGRAPHY
social sciences	MEMORY & COGNITION
health sciences	MOLECULAR AND CELLULAR ENDOCRINOLOGY
biological sciences	MOLECULAR CELL
biological sciences	MOLECULAR ECOLOGY
social sciences	NEW MEDIA & SOCIETY
biological sciences	NUCLEIC ACIDS RESEARCH
social sciences	PERSONNEL PSYCHOLOGY
biological sciences	PLANT CELL
social sciences	POLITICAL ANALYSIS
biological sciences	PROCEEDINGS OF THE ROYAL SOCIETY B-BIOLOGICAL SCIENCES
social sciences	PUBLIC ADMINISTRATION REVIEW
social sciences	PUBLIC CHOICE
health sciences	STROKE
social sciences	STRUCTURAL EQUATION MODELING-A MULTIDISCIPLINARY JOURNAL
