# Supplementary material for: Journal data policies: Exploring how the understanding of editors and authors corresponds to the policies themselves
Source: PLoS One. 2020 Mar 25;15(3):e0230281. doi: 10.1371/journal.pone.0230281 (PMC7094825; doi:10.1371/journal.pone.0230281)
Supplement: S1 Table — (DOCX) [file pone.0230281.s004.docx]

**S1 Table. Editor indication of presence of data policy.**

| **Has the journal issued a policy that requires authors to provide access to data, code, and/or research materials underlying research findings presented in their articles?** | | |
| --- | --- | --- |
|  | **Yes** | **No** |
| **Biological Sciences** (n=43) | 35 (81.4%) | 8 (18.6%) |
| **Health Sciences** (n=20) | 6 (30.0%) | 14 (70.0%) |
| **Social Sciences** (n=51) | 24 (48.0%) | 26 (52%) |
| **Total** (n=113) | **65 (57.5%)** | **48 (42.5%)** |
