# Supplementary material for: Journal data policies: Exploring how the understanding of editors and authors corresponds to the policies themselves
Source: PLoS One. 2020 Mar 25;15(3):e0230281. doi: 10.1371/journal.pone.0230281 (PMC7094825; doi:10.1371/journal.pone.0230281)
Supplement: S2 Table — (DOCX) [file pone.0230281.s005.docx]

**S2 Table. Editor indication of specific transparency requirements.**

|  | **Data**  **transparency** | **Analytic methods transparency** | **Research materials transparency** |
| --- | --- | --- | --- |
| **Biological Sciences** (n=26) | 20 (76.9%) | 14 (53.8%) | 9 (34.6%) |
| **Health Sciences** (n=4) | 1 (25.0%) | 2 (50.0%) | 1 (25.0%) |
| **Social Sciences** (n=21) | 13 (61.9%) | 12 (57.1%) | 9 (42.9%) |
| **Total*** (n=51) | **34 (66.7%)** | **28 (54.9%)** | **19 (37.3%)** |

*The frequency of editors responding to this question is less than the number who reported the presence of a data policy. This is because of editors who either did not answer this question, answered “no” to all options, or who indicated that their journal does not publish empirical research studies.
