# Supplementary material for: Journal data policies: Exploring how the understanding of editors and authors corresponds to the policies themselves
Source: PLoS One. 2020 Mar 25;15(3):e0230281. doi: 10.1371/journal.pone.0230281 (PMC7094825; doi:10.1371/journal.pone.0230281)
Supplement: S3 Table — (DOCX) [file pone.0230281.s006.docx]

**S3 Table. Editor indication of reproducibility verification requirement.**

|  | **Verification** |
| --- | --- |
| **Biological Sciences** (n=26) | 1 (3.8%) |
| **Health Sciences** (n=4) | 0 (0.0%) |
| **Social Sciences** (n=21) | 5 (23.8%) |
| **Total** (n=51) | **6 (11.8%)** |
