# Supplementary material for: Journal data policies: Exploring how the understanding of editors and authors corresponds to the policies themselves
Source: PLoS One. 2020 Mar 25;15(3):e0230281. doi: 10.1371/journal.pone.0230281 (PMC7094825; doi:10.1371/journal.pone.0230281)
Supplement: S4 Table — (DOCX) [file pone.0230281.s007.docx]

**S4 Table**. **Authors’ reported ease or difficulty in understanding journal-issued data policy requirements.**

| **How easy or difficult was it for you to understand what was expected of you to fulfill the requirements of the data policy?** | | | | |
| --- | --- | --- | --- | --- |
|  | **Very**  **easy** | **Somewhat easy** | **Somewhat difficult** | **Very**  **difficult** |
| **Biological Sciences** (n=42) | 17 (40.5%) | 20 (47.6%) | 5 (11.9%) | 0 (0.0%) |
| **Health Sciences** (n=1) | 0 (0.0%) | 1 (100.0%) | 0 (0.0%) | 0 (0.0%) |
| **Social Sciences** (n=30) | 13 (43.3%) | 12 (40.0%) | 4 (13.3%) | 1 (3.3%) |
| **Total** (n=73) | **30 (41.1%)** | **33 (45.2%)** | **9 (12.3%)** | **1 (1.4%)** |
