# Supplementary material for: Journal data policies: Exploring how the understanding of editors and authors corresponds to the policies themselves
Source: PLoS One. 2020 Mar 25;15(3):e0230281. doi: 10.1371/journal.pone.0230281 (PMC7094825; doi:10.1371/journal.pone.0230281)
Supplement: S5 Table — (DOCX) [file pone.0230281.s008.docx]

**S5 Table.** **Author understanding of policy journal-issued data policy requirements.**

|  | **Data**  **Transparency** | **Analytic Methods Transparency** | **Research Materials Transparency** |
| --- | --- | --- | --- |
| **Biological Sciences** (n=18) | 15 (83.3%) | 7 (38.9%) | 5 (27.8%) |
| **Health Sciences** (n=1) | 0 (0.0%) | 0 (0.0%) | 0 (0.0%) |
| **Social Sciences** (n=15) | 8 (53.3%) | 9 (60.0%) | 8 (53.3%) |
| **Total*** (n=34) | **23 (67.6%)** | **16 (47.1%)** | **13 (38.2%)** |

* In cases where more than one author for a journal responded to the survey, we report the presence of the transparency requirement where at least one author confirmed its presence.
