# Supplementary material for: Journal data policies: Exploring how the understanding of editors and authors corresponds to the policies themselves
Source: PLoS One. 2020 Mar 25;15(3):e0230281. doi: 10.1371/journal.pone.0230281 (PMC7094825; doi:10.1371/journal.pone.0230281)
Supplement: S6 Table — (DOCX) [file pone.0230281.s009.docx]

**S6 Table.** **Location of data policy text.**

|  | **Embedded** | **Indexed** | **Dedicated webpage** | **Document download** | **No policy found** |
| --- | --- | --- | --- | --- | --- |
| **Biological Sciences** (n=26) | 13 (50.0%) | 9 (34.6%) | 2 (7.7%) | 0 (0.0%) | 2 (7.7%) |
| **Health Sciences** (n=4) | 2 (50.0%) | 2 (50.0%) | 0 (0.0%) | 0 (0.0%) | 0 (0.0%) |
| **Social Sciences** (n=21) | 10 (47.6%) | 6 (28.6%) | 1 (4.8%) | 2 (9.5%) | 2 (9.5%) |
| **Total*** (n=51) | **25 (49.0%)** | **17 (33.3%)** | **3 (5.9%)** | **2 (3.9%)** | **4 (7.8%)** |
