# Supplementary material for: Journal data policies: Exploring how the understanding of editors and authors corresponds to the policies themselves
Source: PLoS One. 2020 Mar 25;15(3):e0230281. doi: 10.1371/journal.pone.0230281 (PMC7094825; doi:10.1371/journal.pone.0230281)
Supplement: S7 Table — (DOCX) [file pone.0230281.s010.docx]

**S7 Table.** **Mention of transparency requirements in policy language.**

|  | **Data**  **Transparency** | **Analytic Methods Transparency** | **Research Materials Transparency** |
| --- | --- | --- | --- |
| **Biological Sciences** (n=24) | 24 (100.0%) | 20 (76.9%) | 17 (65.4%) |
| **Health Sciences** (n=4) | 4 (100.0%) | 4 (100.0%) | 3 (75.0%) |
| **Social Sciences** (n=19) | 19 (100.0%) | 14 (75.0%) | 13 (70.0%) |
| **Total*** (n=47) | **47 (100.0%)** | **38 (73.7%)** | **33 (70.2%)** |

*The number of policies is less than the number for which editors indicated a policy is in place because we were unable to locate policies for 4 journals.
