# Supplementary material for: Journal data policies: Exploring how the understanding of editors and authors corresponds to the policies themselves
Source: PLoS One. 2020 Mar 25;15(3):e0230281. doi: 10.1371/journal.pone.0230281 (PMC7094825; doi:10.1371/journal.pone.0230281)
Supplement: S8 Table — (DOCX) [file pone.0230281.s011.docx]

**S8 Table. Excerpts from policy text used to establish encouragement or requirement of policy conditions.**

| **Encouragement** | **Requirement** |
| --- | --- |
| In general, you **should**…  Authors **are invited to**…  Authors are **generally expected to**...  ...authors **are encouraged to**…  Authors are **strongly encouraged to**...  ...is **not strictly a requirement**…  ...authors **are asked to**…  ...it is **at your discretion to decide**…  We **recommend** that you…  ...is the journal’s **preferred option**. | …authors **are required to**...  An **implicit term and condition** of publishing...  ...authors of accepted papers **must**…  ...**requires, as a condition** for publication…  The policy...is to publish papers **only if**…  ...**is mandatory** for publication…  All manuscripts...**will be subjected to**… |
