# Supplementary material for: Journal data policies: Exploring how the understanding of editors and authors corresponds to the policies themselves
Source: PLoS One. 2020 Mar 25;15(3):e0230281. doi: 10.1371/journal.pone.0230281 (PMC7094825; doi:10.1371/journal.pone.0230281)
Supplement: S9 Table — (DOCX) [file pone.0230281.s012.docx]

**S9 Table.**  **Stringency of transparency requirements as expressed in policy language.**

|  | **Encourage** | **Require** | **Encourage + Require** |
| --- | --- | --- | --- |
| **Biological Sciences** (n=24) | 2 (8.3%) | 11 (45.8%) | 11 (45.8%) |
| **Health Sciences** (n=4) | 2 (50.0%) | 1 (25.0%) | 1 (25.0%) |
| **Social Sciences** (n=19) | 6 (31.6%) | 10 (52.6%) | 2 (10.5%) |
| **Total** (n=47) | **10 (21.3%)** | **22 (46.8%)** | **14 (29.8%)** |
