# Supplementary material for: Journal data policies: Exploring how the understanding of editors and authors corresponds to the policies themselves
Source: PLoS One. 2020 Mar 25;15(3):e0230281. doi: 10.1371/journal.pone.0230281 (PMC7094825; doi:10.1371/journal.pone.0230281)
Supplement: S10 Table — (DOCX) [file pone.0230281.s013.docx]

**S10 Table. Examples of terms used in policies to refer to categories of transparency requirements.**

| **Data** | **Analytic Methods** | **Research Materials** |
| --- | --- | --- |
| data  data set  microarray data  sequence data  genetically modified organisms and mutants  electron microscopy data  genotype data  nucleotide sequences  proteins sequence data  raw data  certain types of data  stimulus norms  supporting data | protocols  programs  computer code  computer programs  scripts  methods  program code  software  algorithms  models  statistical tools  analytic methods  laboratory protocols  commands | materials  other details  description  readme file  additional information  related materials  metadata  other useful materials  other artifacts  explanatory file  codebook  relevant description |
